# Sphingolipids: towards an integrated view of metabolism during the plant stress response

**DOI:** 10.1111/nph.15997

**Published:** 2019-07-15

**Authors:** Eloïse Huby, Johnathan A. Napier, Fabienne Baillieul, Louise V. Michaelson, Sandrine Dhondt‐Cordelier

**Affiliations:** ^1^ Résistance Induite et Bioprotection des Plantes EA 4707 SFR Condorcet FR CNRS 3417 University of Reims Champagne‐Ardenne BP 1039 F‐51687 Reims Cedex 2 France; ^2^ Laboratoire de Biophysique Moléculaire aux Interfaces Gembloux Agro‐Bio Tech Université de Liège 2 Passage des Déportés B‐5030 Gembloux Belgique; ^3^ Plant Sciences Rothamsted Research West Common Harpenden AL5 2JQ UK

**Keywords:** abiotic stress, biotic stress, pathogens, plant defence, programmed cell death, sphingolipid

## Abstract

Plants exist in an environment of changing abiotic and biotic stresses. They have developed a complex set of strategies to respond to these stresses and over recent years it has become clear that sphingolipids are a key player in these responses. Sphingolipids are not universally present in all three domains of life. Many bacteria and archaea do not produce sphingolipids but they are ubiquitous in eukaryotes and have been intensively studied in yeast and mammals. During the last decade there has been a steadily increasing interest in plant sphingolipids. Plant sphingolipids exhibit structural differences when compared with their mammalian counterparts and it is now clear that they perform some unique functions. Sphingolipids are recognised as critical components of the plant plasma membrane and endomembrane system. Besides being important structural elements of plant membranes, their particular structure contributes to the fluidity and biophysical order. Sphingolipids are also involved in multiple cellular and regulatory processes including vesicle trafficking, plant development and defence. This review will focus on our current knowledge as to the function of sphingolipids during plant stress responses, not only as structural components of biological membranes, but also as signalling mediators.

## Introduction

The strategies that plants employ to endure stressful conditions are varied and involve a multitude of molecular, metabolic and physiological adaptations. There is now a significant body of work to indicate that sphingolipids are an important part of the arsenal of tools the plant has at its disposal to respond to stress. Sphingolipids are an incredibly diverse group of compounds (Pata *et al*., 2010) with a vast array of physical properties that facilitate their function in a variety of cellular processes. Sphingolipids form a significant proportion of the lipids present in higher plants. Studies suggest sphingolipids constitute up to 40% of lipids in the plasma membrane of plant cells (Cacas *et al*., 2016) and are enriched in the endosomes and tonoplasts (Moreau *et al*., 1998). More comprehensive extraction techniques have been developed over recent years that, when coupled with technological advances in mass spectrometry and chromatography, have allowed improved sphingolipid identification and the discovery of novel structures from smaller quantities of material (Cacas *et al*., 2016). This situation has enabled researchers to determine the contribution that sphingolipid metabolites make in different cellular processes.

**Figure 1:**
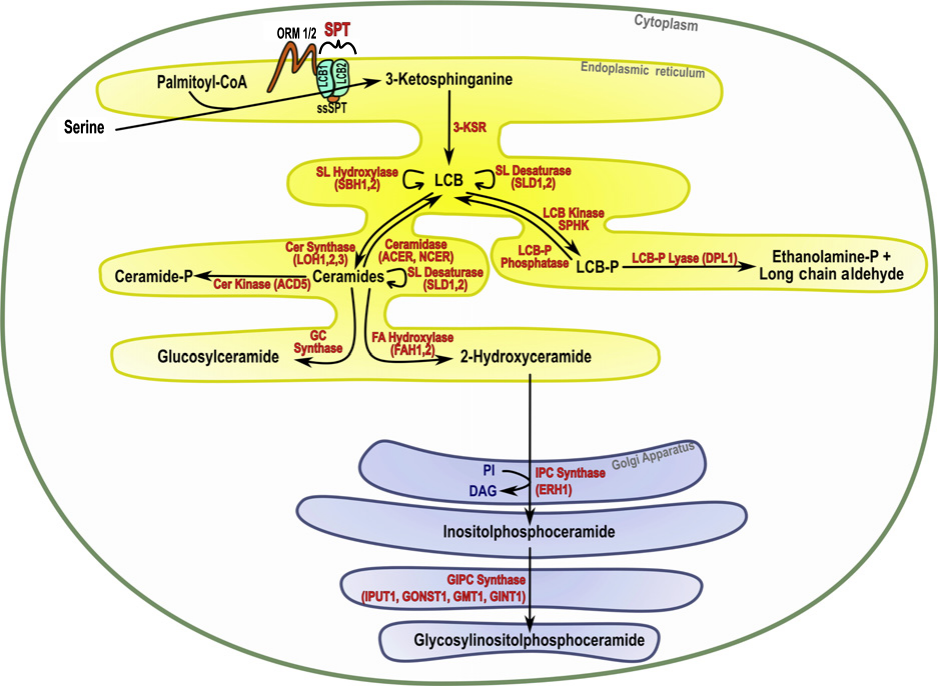
Schematic representation of the sphingolipid biosynthetic pathway in plants. 3‐KSR, 3‐ketosphinganine reductase; ACD5, accelerated cell death 5; ACER, alkaline ceramidase; Cer, ceramide; ceramide‐P, ceramide‐phosphate; CoA, coenzyme A; DAG, diacylglycerol; DPL1, dihydrosphingosine phosphate lyase; ERH1, enhancing RPW8‐mediated HR‐like cell death; FA, fatty acid; FAH, fatty acid hydroxylase; GC, glucosylceramide; GINT1, glucosamine inositol phosphorylceramide transferase 1; GIPC, glycosyl inositol phosphoceramide; GMT1, GIPC mannosyl‐transferase 1; GONST1, Golgi localized nucleotide sugar transporter 1; IPC, inositol phosphorylceramide; IPUT, inositol phosphorylceramide glucuronosyltransferase 1; LCB1,2, subunit of serine palmitoyltransferase 1 and 2; LCB, long‐chain base; LCB‐P, long‐chain base phosphate; LOH, LAG1 homolog; NCER, neutral ceramidase; ORM, orosomucoid‐like protein; PI, phosphoinositol; SBH, sphingoid base hydroxylase; SL, sphingolipid; SLD, sphingolipid Δ8 long‐chain base desaturase; SPHK, sphingosine kinase; ssSPT, small subunit of serine palmitoyl transferase; SPT, serine palmitoyl transferase.

An overview of the sphingolipid biosynthetic pathway is presented in Fig. 1. The term sphingolipid covers a class of lipids whose defining component is a long‐chain or sphingoid base (LCB; for ease of reference, Supporting Information Table S1 lists the abbreviations used in this review). The LCB is a carbon amino‐alcohol backbone most commonly of 18 carbons that is synthesised by the condensation of serine and palmitoyl‐CoA catalysed by serine palmitoyl transferase (SPT) in the endoplasmic reticulum (ER) (Chen *et al*., 2006). The product of this reaction, 3‐ketosphinganine, is then reduced by the action of the 3‐ketosphinganine reductase to sphinganine (d18:0) (Beeler *et al*., 1998). The LCB is considered the simplest functional sphingolipid and can have a range of modifications including phosphorylation, desaturation and hydroxylation. It is sometimes referred to as the free LCB. The LCB may be linked to a very‐long‐chain fatty acid *via* an amide bond to form a ceramide. The fatty acyl component is usually 16–26 carbons. This reaction is catalysed by ceramide synthase. In *Arabidopsis thaliana* (hereafter Arabidopsis) three ceramide synthases have been identified, LOH1–3. Ceramidases catalyse the reverse reaction and are a component in regulating the ceramide pool and sphingolipid homeostasis (Pata *et al*., 2008). Ceramides can be phosphorylated in the endoplasmic reticulum (ER) by ceramide kinases (CerK) or ACD5 (accelerated cell death 5) or further modified to form the complex sphingolipids glycosylceramides (GlcCers) in the ER and glycosyl inositol phosphorylceramides (GIPCs) by the addition of simple or multiple sugars on ceramide at the C1 position in the Golgi. These reactions are catalysed by glucosylceramide synthase (GCS) and at least three functional IPC‐synthases and several glycosyl or glucuronyl transferases (Wang *et al*., 2008; Mina *et al*., 2010; Rennie *et al*., 2014; Msanne *et al*., 2015). The complex sphingolipids can exhibit very high levels of sugar decoration. One study of 23 plant species identified at least 21 different patterns showing variation in number, type and order of glycan substitutions (Cacas *et al*., 2013). The biosynthesis of complex sphingolipids is tightly controlled and the GIPC pool is regulated by the hydrolysis of GIPC to phytoceramide‐1 phosphate by the action of a phospholipase D (PLD) (Tanaka *et al*., 2013). Functional characterisations of enzymes of the sphingolipid biosynthetic pathway have also pointed to the controls on the pathway and the specific pool sizes and structures that are generated. This flexibility enables sphingolipids to constitute both a structural membrane component and a signalling molecule from the same basic lipid backbones. For more details about sphingolipid biosynthesis, see the recent reviews by Luttgeharm *et al*., 2016; Michaelson *et al*., 2016 and Mamode Cassim *et al*., 2019.

In plants, the size of the different sphingolipid pools tends to vary in a species‐specific and tissue‐dependent manner. For example, the occurrence of the LCB d18:2 containing GlcCer in Arabidopsis is mainly confined to floral and pollen tissue (Michaelson *et al*., 2009) and sphingolipid distribution changes during fruit development and ripening (Ines *et al*., 2018). However outside the Brassicaceae family d18:2 production occurs throughout the plant and, in species such as tomato and soybean, it is the most abundant GlcCer (Markham *et al*., 2006). Wheat was found to contain much higher levels of d18:1 in its LCBs when compared with rice (Goto *et al*., 2012). In addition, the different tissues in rice have been found to contain a similar quantity of sphingolipids, but distribution across the lipid classes was altered. A survey of 21 different plant species from different phylogenetic groups found d18:1^Δ4^ to be present in nonseed land plants and monocots but absent from Arabidopsis and soybean (Islam *et al*., 2012).

The functional significance of variations in sphingolipid chemical diversity and abundance is still in the early stages of investigation. The different classes and modifications offer a variety of differing solubility, charge, shape and size. It is this array of properties that confers the potential of sphingolipids to function both as bio‐active components of cells involved in regulating cellular processes and as integral components involved in the structural integrity of the membranes. Regulation of sphingolipid metabolism enables plants to facilitate cell growth and to appropriately respond to stress, both biotic and abiotic, using different metabolites to modulate its response.

Here, we summarise our current knowledge on the role of sphingolipids in plants in response to environmental cues and stress.

## Signals in programmed cell death

Recent work utilising genetically altered plants and plants exposed to sphingolipid biosynthesis inhibitors have revealed that sphingolipids are regulators of programmed cell death (PCD) occurring either during plant development or immunity. Perception of a stress often occurs at the plasma membrane level. Therefore its integrity is essential for cell signalling and survival. Sphingolipids are major structural constituents of plant plasma membrane microdomains and their relationship with other components of the plasma membrane is crucial. Changes in sphingolipid biosynthesis therefore affect the microdomain composition and this could affect protein content and distribution due to altered interactions between plasma membrane components. For example, Bax‐inhibitor‐1 (AtBI‐1, an inhibitor of Bax‐induced cell death) interacts with both FAH1 and FAH2 (fatty acid 2‐hydroxylase). Plants overexpressing AtBI‐1 therefore displayed enrichment in 2‐hydroxy fatty acid‐containing GlcCer in microdomains as well as a loss of two proteins that are usually specifically localised to microdomains (Ishikawa *et al*., 2015). These two proteins feature in plant defence, both being involved in cell death triggered by salicylic acid (SA) or oxidative stress. This reduction in protein content led to an enhanced tolerance to SA or oxidative stress in AtBI‐1‐overexpressing plants (Ishikawa *et al*., 2015). These data suggest that the integrity of microdomains is critical to cell death and sphingolipids are central to these structures.

**Figure 2:**
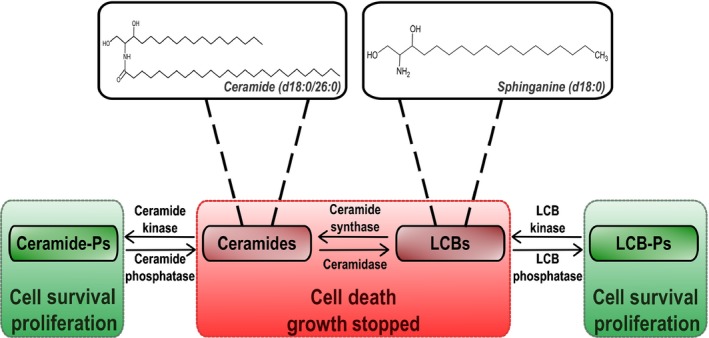
Sphingolipid rheostat. The equilibrium between ceramides/long‐chain bases (LCBs) and ceramide phosphates (ceramide‐Ps)/LCB‐Ps defines cell fate.

Sphingolipids are involved in the control of PCD, either as structural components of membranes but also as initiators in the cell death regulatory pathway. The existence of a rheostat between ceramides/LCBs and their phosphorylated counterparts, already described in animal cells, is thought to exist in plants and similarly to control cell fate. According to this model, ceramides and LCBs are able to trigger cell death, whereas ceramide phosphates and LCB‐Ps promote cell survival (Shi *et al*., 2007; Alden *et al*., 2011) (Fig. 2). The induction of PCD by LCB was based on the activation of protein kinases, MPK6 (Saucedo‐Garcia *et al*., 2011) or 14‐3‐3‐regulated CPK3 (Lachaud *et al*., 2013). The spontaneous PCD observed in the *acd5* mutant, defective in ceramide kinase and with enhanced levels of ceramides, was due to a strong accumulation of mitochondrial reactive oxygen species (ROS) (Bi *et al*., 2014). This finding suggests that ROS are a component of sphingolipid‐induced PCD. The mycotoxin fumonisin B1 (FB1) has been widely used to study both sphingolipid biosynthesis and PCD. Indeed, FB1 is a strong inhibitor of ceramide synthase and has been shown to induce PCD. When applied to plants, FB1 also triggered the accumulation of LCBs and LCB‐Ps (Shi *et al*., 2007; Tsegaye *et al*., 2007; Saucedo‐Garcia *et al*., 2011; Yanagawa *et al*., 2017). Overexpression of *AtLCBK1* (Arabidopsis sphingoid LCB kinase) in a recent study in plants induced resistance to FB1 treatment and, conversely, *AtLCBK1* knockdown plants exhibited a sensitivity to such a treatment (Yanagawa *et al*., 2017). Moreover, the authors demonstrated that transgenic alteration of proteins involved in LCB/LCB‐P homeostasis (AtLCBK1, AtSPP1 and AtDPL1) resulted in a positive correlation between the levels of free LCBs and the degree of FB1‐induced cell death (Yanagawa *et al*., 2017).

Increase in SPT activity, by overexpression of *AtssSPTa* (small subunit of SPT), resulted in an accumulation of LCBs and reduced tolerance to FB1, whereas *AtssSPTa* suppression lines displayed lower levels of LCBs but enhanced tolerance to FB1 (Kimberlin *et al*., 2013). It was recently demonstrated by two independent studies that orosomucoid‐like proteins AtORM1 and AtORM2 physically interact with the core SPT complex and function as a repressor of SPT activity (Kimberlin *et al*., 2016; Li *et al*., 2016). ORM proteins therefore regulate sphingolipid homeostasis by differently modulating functionally different ceramide synthase activities (Kimberlin *et al*., 2016). *AtORM1* and *AtORM2* overexpressing plants were more tolerant to FB1 treatment when compared with wild‐type (WT) plants. This tolerance is accompanied by a lower accumulation of C16 ceramides, LCBs and their phosphorylated counterparts. Conversely, *AtORM* RNAi lines were more sensitive to such treatment, and displayed higher content of C16 ceramides, LCBs and LCB‐Ps (Kimberlin *et al*., 2016). Similarly, the ceramide synthase *LOH2* overexpressing lines resulted in the accumulation of ceramides containing C16 fatty acids and dihydroxy LCBs and had reduced accumulation of free LCBs and LCB‐Ps in response to FB1. This overexpression also resulted in constitutive induction of PCD and increased resistance to FB1 (Luttgeharm *et al*., 2015). These findings suggested that FB1‐induced PCD is primarily due to the accumulation of free LCBs rather than the accumulation of ceramides containing C16 fatty acids/dihydroxy LCBs. Curiously, growth and increased cell division were promoted in *LOH1* and *LOH3* overexpressing plants, which displayed enhanced production of ceramides with very‐long‐chain fatty acids (VLCFAs) and trihydroxy LCBs (Luttgeharm *et al*., 2015). These unexpected outcomes for growth and development could be due to a ceramide synthesis with a certain chain length fatty acid and quantity and in response to the correct stimuli. It is also known that VLCFA ceramides are important for Golgi trafficking and cell plate or phragmoplast formation during cell division in Arabidopsis (Molino *et al*., 2014). It is therefore possible that increased cell expansion could be due to sphingolipid targeting to plant membranes that contributes directly to cell expansion. In addition, the fatty acid hydroxylase double mutant *fah1*/*fah2* fails to form spontaneous lesions under standard culture conditions, despite an accumulation in free trihydroxy LCBs, C16 ceramides and VLCFA ceramides and SA (König *et al*., 2012). Moreover, the *gonst1* (Golgi localised nucleotide sugar transporter1, involved in glycosylation of GIPCs) mutant displayed spontaneous hypersensitive reaction (HR)‐like lesions but did not accumulate ceramides or LCBs (Mortimer *et al*., 2013). One potential explanation for these observed differences is that several different mechanisms could be responsible for inducing cell death.

## Sphingolipids as structural components in response to abiotic stress

Several studies have recently reported a role for sphingolipids in response to temperature stress. Acclimation capacity was correlated with changes in the content of TAGs (triacylglycerols), MGDG (monogalactosyldiacylglycerol), DGDG (digalactosyldiacylglycerol) and a GlcCer (Degenkolbe *et al*., 2012). Analysis of oat, rye and Arabidopsis lipid profiles during cold acclimation demonstrated that GlcCer contents decreased in the plasma membrane, whereas they were unchanged in microdomains (Minami *et al*., 2009; Takahashi *et al*., 2016). These changes could contribute to a greater hydration of the plasma membrane that could, in turn, increase membrane stability during cold stress. In a study focusing on grapevine leaves, it was found that high levels of t18:1 (8Z) in complex sphingolipids were correlated with freezing tolerance (Kawaguchi *et al*., 2000). The sphingolipid Δ8 long‐chain base desaturases (SLD), which desaturate the LCB at the Δ8 position in both *cis* and *trans* orientations, appear to play a role in cold tolerance in Arabidopsis (Chen *et al*., 2012) and tomato (Zhou *et al*., 2016). In Arabidopsis, the *sld1sld2* double mutant is sensitive to cold stress (Chen *et al*., 2012). Similarly, *SlSLD* knockdown tomato plants displayed greater membrane damage and physiological indicators of chilling damage after stress than WT plants. Chloroplasts are the main organelle affected by cold and many studies have reported that chloroplast morphology is affected by changes in lipid unsaturation. Chloroplasts in *SlSLD* knockdown were more severely damaged than in WT plants and the surviving organelles were not surrounded by an extra membrane (Zhou *et al*., 2016). GlcCers, believed to stabilise membranes, were detected in the envelope membrane of chloroplasts (Spassieva & Hille, 2003), suggesting that sphingolipids are structurally important for chloroplast membrane for cold tolerance. This finding illustrated that disrupting *SlSLD* transcript accumulation reduced chilling tolerance of tomato. Lipid desaturation is a way for plants to mitigate the effects of chilling or freezing temperatures. *SlSLD* knockdown plant sensitivity to chilling could therefore be related to membrane properties such as fluidity, which is diminished due to depletion of sphingolipids with unsaturated LCBs. Another explanation for the decrease in cold tolerance could be a change in the formation and content of microdomains in the membrane. It is conceivable that activity of some microdomain‐localised proteins important for cold tolerance could be modified in perturbed microdomains (Chen *et al*., 2012). There has been no characterised function for sphingolipids in tolerance of high temperature by contrast with the high concentration of trienoic fatty acids in the thylakoid membranes that have been shown to be involved in both chilling and high temperature tolerance (Murakami *et al*., 2000; Routaboul *et al*., 2012; Tovuu *et al*., 2016).

## Sphingolipids as structural components in response to biotic stress

The rice *Osfah1/2* plants displayed similar SA levels to WT and a decreased tolerance to the hemibiotrophic fungus *Magnaporthe oryzae*. Nagano and colleagues demonstrated that products of these enzymes, 2‐hydroxy‐sphingolipids, were critical in the formation of microdomains and disruption of OsFah1/2 activity disturbed organisation of defence proteins localized in these microdomains, such as the NADPH oxidase RbohB, required for ROS production involved in rice immunity (Nagano *et al*., 2016).

Recent work has identified three genes involved in GIPC glycosylation: GONST1, IPUT1 (inositol phosphorylceramide glucuronosyltransferase1) and GMT1 (GIPC mannosyl‐transferase1) (Mortimer *et al*., 2013; Fang *et al*., 2016; Tartaglio *et al*., 2017). These three mutants displayed high SA and ROS levels coupled to a constitutive HR and defence‐gene induction, suggesting a constitutive biotic stress response. Interestingly, *gmt1* also had a decrease in cellulose accompanied by an increase in lignin content, a well known process in disease resistance.

Eudicot plant‐specific GIPCs appeared to act as NLP (necrosis and ethylene‐inducing peptide 1‐like protein) cytolysin receptors (Lenarcic *et al*., 2017). NLP are produced by bacterial, fungal and oomycete plant pathogens. Monocots did not develop necrotic lesions upon challenge with NLP. The difference between the two clades resides in the length of terminal hexose residues in GIPCs (two for eudicots and three for monocots). The GIPC sugar moiety is exposed at the surface of the plasma membrane and is therefore accessible to NLP binding. The presence of a third hexose unit in monocots impeded NLP insertion into the plasma membrane. The structural and molecular consequences for the plasma membrane that could occur downstream of this recognition requires further study. These studies demonstrate that GIPC glycosylation and the identity of the glycan headgroup are important for the plant immune response.

## Sphingolipids as signalling messengers in abiotic stress

The sessile nature of plants has driven them to develop a myriad of strategies to resist cell damage. Abiotic stress affects plant growth and development, resulting in loss of vigour and ultimately death. The altered physical and chemical composition of cell membranes under temperature, salt stress or hypoxia is a problem the plant must manage. As a major component of plasma membranes, sphingolipids are significant in mitigating abiotic stress, both in plasma membrane remodelling, and as signal transduction molecules (Ali *et al*., 2018). A summary of the available data on the enzymes and genes of the sphingolipid pathway involved in response to both abiotic and biotic stress is presented in Table 1.

**Table 1:** Enzymes and genes of sphingolipid metabolism involved in response to (a)biotic stress.

Enzyme	Name	Mutant/transgenic plants	Phenotype under (a)biotic stress	References
Sphingolipid ∆8 long‐chain base desaturases	SLD	*sld1sld2* (Arabidopsis)	Sensitive to cold	Chen *et al*. (2012)
		*SlSLD‐*KD (tomato)	Sensitive to chilling	Zhou *et al*. (2016)
Long‐chain base kinase	LCBK1	*lcbk1* (Arabidopsis)	Freezing tolerant	Huang *et al*. (2017)
		*lcbk1‐*KD (Arabidopsis)	Sensitive to FB1 treatment	Yanagawa *et al*. (2017)
		*OsLCBK1*‐OE (rice)	Tolerance to oxidative stress	Zhang *et al*. (2013)
		*AtLCBK1*‐OE (Arabidopsis)	Tolerance to FB1 treatment	Yanagawa *et al*. (2017)
Long‐chain base kinase	LCBK2	*lcbk2* (Arabidopsis)	Tolerance to intermediate cold (12°C)	Dutilleul *et al*. (2012)
Long‐chain base kinase	SPHK1	*SPHK1*‐OE (Arabidopsis)	Sensitive to ABA treatment	Worrall *et al*. (2008)
Ceramide kinase	ACD5	*acd5* (Arabidopsis)	Seed germination sensitive to cold	Dutilleul *et al*. (2015)
			Tolerance to powdery mildew	Wang *et al*. (2008)
			Susceptibility to *B. cinerea*	Bi *et al*. (2014)
Ceramide synthase	LOH1LOH2LOH3	*loh1, loh2, loh3* (Arabidopsis)	Sensitivity to dark submergence	Xie *et al*. (2015a)
		*loh1‐1 loh3‐1* (Arabidopsis)	Sensitivity to dark and light submergence	Xie *et al*. (2015a)
		*LOH2‐*OE (Arabidopsis)	Tolerance to FB1 treatment	Luttgeharm *et al*. (2015)
Neutral ceramidase	nCER1	*ncer1* (Arabidopsis)	Sensitivity to oxidative stress	Li *et al*. (2015)
		*nCer1*‐OE (Arabidopsis)	Tolerance to oxidative stress	Li *et al*. (2015)
Alkaline ceramidase	AtACER	*Atacer* (Arabidopsis)	Sensitivity to oxidative stress	Zheng *et al*. (2018)
			Susceptibility to *P. syringae* strain DG3	Wu *et al*. (2015a)
		*Atacer, AtACER* RNAi (Arabidopsis)	Sensitivity to salinity	Wu *et al*. (2015a)
		*AtACER*‐OE (Arabidopsis)	Tolerance to salinity	Wu *et al*. (2015a)
Sphingosine‐1 phosphate lyase	OsSPL1	*OsSPL1*‐OE (rice)	Sensitivity to salinity	Zhang *et al*. (2012)
			Susceptibility to *P. syringae* pv. *tabaci*	Zhang *et al*. (2014)
Sphingoid phosphate phosphatase1	AtSPP1	*Atssp1* (Arabidopsis)	Sensitive to ABA treatment	Nakagawa *et al*. (2012)
Dihydrosphingosine‐1‐phosphate lyase1	AtDPL1	*Atdpl1* (Arabidopsis)	Susceptibility to *P. syringae* pv. *tomato* and tolerant to *B. cinerea*	Magnin‐Robert *et al*. (2015)
Fatty acid alpha‐hydroxylase	FAH1FAH2	*fah1/fah2* (Arabidopsis)	Tolerance to powdery mildew	König *et al*. (2012)
		*OsFah1/OsFah2* (rice)	Susceptibility to *Magnaporthe oryzae*	Nagano *et al*. (2016)
Enhancing RPW8‐mediated HR‐like cell death	ERH1	*erh1* (Arabidopsis)	Tolerance to powdery mildew	Wang *et al*. (2008)
Glucosamine inositol phosphorylceramide transferase1	AtGINT1	*Atgint1* (Arabidopsis)	Tolerance to moderate salinity	Ishikawa *et al*. (2018)
Serine palmitoyltransferase	SPT	*SPT*‐silenced (tobacco)	Susceptibility to *Alternaria alternata* f. sp. *lycopersici*	Rivas‐San Vicente *et al*. (2013)
Small subunit of serine palmitoyltransferase	ssSPTa	*AtssSPTa*‐OE (Arabidopsis)	Sensitivity to FB1 treatment	Kimberlin *et al*. (2013)
		*AtssSPTa* RNAi (Arabidopsis)	Tolerance to FB1 treatment	Kimberlin *et al*. (2013)
Subunit of serine palmitoyltransferase	LCB2a1	*OsLCB2a*‐OE (rice)	Tolerance to *Myzus persicae* infestation	Begum *et al*. (2016)
Orosomucoid‐like proteins	ORM1ORM2	*orm1* amiR‐*ORM2* (Arabidopsis)	Tolerance to *P. syringae* strain DG3	Li *et al*. (2016)
			Tolerance to oxidative stress	Li *et al*. (2016)
		*AtORM1*‐OE, *AtORM2*‐OE (Arabidopsis)	Tolerance to FB1 treatment	Kimberlin *et al*. (2016)
		*AtORM1* RNAi, *AtORM2* RNAi(Arabidopsis)	Sensitivity to FB1 treatment	Kimberlin *et al*. (2016)

KD, knocked‐down; OE, overexpressing line.

### Temperature stress

Sphingolipids are involved in cold acclimation as structural components of membranes and also as signalling molecules. In Arabidopsis WT plants, low temperatures trigger an accumulation of total sphingolipids, whereas the ratio of unsaturated LCBs is not increased by low temperatures (Nagano *et al*., 2014). This situation suggests that sphingolipids containing unsaturated LCBs are potential candidates for natural resistance to low temperatures but not for induced tolerance to cold. The cell death suppressor AtBI‐1 is involved in sphingolipid synthesis in response to cold by interacting with AtSLD1, AtFAH1, AtSBH2 (a LCB C‐4 hydroxylase) and AtADS2 (acyl lipid desaturase 2) through Arabidopsis cytochrome b_5_ (Nagano *et al*., 2014). Moreover, chilling induced a decrease in LCB production (especially t18:1) (Guillas *et al*., 2013). An Arabidopsis mutant exhibiting low levels of nitric oxide (NO) displayed an accumulation of t18:1. A rapid and transient production of t18:0‐P and ceramide phosphates is induced by cold. This accumulation was negatively regulated by NO (Cantrel *et al*., 2011) and was specifically impaired in *lcbk2* (but not in *lcbk1*) or *acd5* mutants, respectively (Dutilleul *et al*., 2012, 2015). Whether NO is able to directly regulate enzymes involved in LCB/LCB‐P and Cer/Cer‐P rheostat or their substrate availability is still unknown. *lcbk2* displayed a constitutive activation of a cold‐responsive MAPK, AtMPK6, at 22°C. AtMPK6 activation was also stimulated by t18:0‐P treatment (Dutilleul *et al*., 2012). The expression of some cold‐responsive genes and phenotypical cold responses were impaired in the *lcbk2* mutant but not in *acd5*. In addition, *acd5* seed germination was hypersensitive to cold and abscisic acid (ABA), however gibberellic acid (GA) treatment reverted the *acd5* germination phenotype at 4°C. Germination is regulated by ABA and GA, two hormones that function antagonistically. This finding suggests that defects in the ABA/GA balance and CerK activity could be responsible for *acd5* seed hypersensitivity (Dutilleul *et al*., 2015). Therefore, some responses are regulated by phosphorylated sphingolipids, ABA and NO signalling during cold stress. Recent data have described a role for LCBK1 in Arabidopsis freezing tolerance (Huang *et al*., 2017). Typical responses including osmolyte accumulation, induction of cold‐ and membrane lipid‐related genes occurring during this abiotic stress are all impaired in the *lcbk1* mutant. This situation suggested a fine‐tuned regulation in which LCBK1 acts as a signal in response to freezing temperatures and LCBK2 in response to chilling temperatures.

There are only a small number of studies indicating that sphingolipid metabolism is also involved in heat stress. It was shown that exogenous LCB‐phosphate contributed to heat stress tolerance in Arabidopsis cell culture (Alden *et al*., 2011). Moreover, a recent transcriptome analysis showed that *AtSLD1* expression is significantly decreased in response to a combination of heat wave and drought at ambient and elevated CO_2_, mimicking global changes in climate (Zinta *et al*., 2018).

### Hypoxia and oxidative stress

Hypoxia leads to an increase in ceramides, hydroxyceramides, GlcCers and GIPCs (Xie *et al*., 2015a,b). In hypoxic conditions, GIPCs are elevated in Arabidopsis and increased further in *Atacbp3* (acyl‐CoA binding protein 3), whereas AtACBP3‐overexpressors were hypersensitive to submergence (Xie *et al*., 2015b; Lung & Chye, 2019). Similarly, a reduction of unsaturated VLC‐ceramides in *loh1*,* loh2* and *loh3* mutants due to the disruption of ceramide synthase is accompanied by an enhanced sensitivity to dark submergence. The *loh1‐1 loh3‐1* double mutant displayed a reduction in unsaturated very‐long‐chain (VLC)‐ceramides and impaired tolerance to dark and light submergence. Unsaturated VLC ceramides are therefore seen as defence molecules for plant tolerance to hypoxia (Xie *et al*., 2015a). The mechanism underlying this tolerance involves the modulation of ethylene signalling. These molecules were shown to interact with constitutive triple response1 (CTR1; a negative regulator in ethylene signalling) and to inhibit its kinase activity (Xie *et al*., 2015a) and subsequent ethylene signalling. Furthermore, the hypersensitivity of *loh* mutants to dark submergence was rescued by introduction of the *crt1‐1* mutation that constitutively induces the ethylene response. Overexpression of long‐chain base kinase (OsLCBK1) in tobacco led to an increased tolerance to oxidative stress provoked by treatment with either methyl viologen or H_2_O_2_, accompanied with an induction of oxidative stress‐related gene expression (Zhang *et al*., 2013). *orm1* amiR‐*ORM2* plants exhibited an early senescence phenotype accompanied by ROS production and they displayed higher survival rates to oxidative stress (Li *et al*., 2016). Measurement of sphingolipids showed an increase in LCBs and ceramides and an active vesicular transport that could contribute to the onset of the senescence phenotype and the resistance to oxidative stress. A homolog of human ceramidase, the neutral ceramidase nCer1, was recently characterised. *ncer1* Arabidopsis plants accumulated hydroxyceramides and were more sensitive to oxidative stress. Conversely, *nCer1* overexpressing plants were more tolerant to oxidative stress (Li *et al*., 2015). Loss of AtACER, encoding an alkaline ceramidase, inhibited autophagy and its overexpression stimulated autophagy under oxidative stress (Zheng *et al*., 2018). The *Atacer* mutant is highly sensitive to oxidative stress, whereas the complementation line showed a similar tolerance to this stress as the WT plant (Zheng *et al*., 2018). This result suggests that AtACER improves adaptation to oxidative stress by regulating autophagy.

### Salt stress

During the early stage of salt stress in *Carex rigescens,* an iTRAQ‐based proteome study showed a reduction of the enzyme that catalyses the second step of the biosynthesis of phytosphingosine, 3‐ketosphingosine reductase (KDSR) (Li *et al*., 2017). Based on work performed in yeast where 3‐ketosphinganine reductase suppressed Ca^2+^ sensitivity (Beeler *et al*., 1998), the authors hypothesised that KDSR acts as a suppressor of the calcium signal during salt stress. Seeds of *Atgint1* (glucosamine inositol phosphorylceramide transferase1, responsible for the glycosylation of some GIPCs) mutants displayed a higher germination rate than WT in response to salt stress, although this difference disappeared at higher salt concentrations (Ishikawa *et al*., 2018). The *Atacer* mutant and *AtACER* RNAi lines displayed high ceramide levels but reduced LCBs due to a disruption of an alkaline ceramidase gene (Wu *et al*., 2015a). Whereas these plants showed increased sensitivity to salinity, *AtACER* overexpression led to an increased tolerance to such a stress, highlighting the involvement of ceramides in response to salt stress. More precisely, it has recently been shown that AtACER regulates autophagy induced by high salt stress (Zheng *et al*., 2018). Overexpression of a rice S1P (sphingosine‐1‐phosphate) lyase gene in tobacco led to a decrease in tolerance to salt and changes in salt stress related genes (Zhang *et al*., 2012). By contrast, overexpression of *OsLCBK1* in tobacco plants triggered no alteration in expression of salt stress‐related genes or tolerance/sensitivity phenotype compared with control plants in response to salt stress (Zhang *et al*., 2013), suggesting that this enzyme is not involved in salt stress responses in rice. Bioinformatic analysis supported the hypothesis that there are at least two OsLCBKs (Zhang *et al*., 2013). No sphingolipidomic analysis has been performed to reveal how the LCB content could vary between these two overexpressing plants. Previously published papers suggested that the sphingolipid metabolism could be adjusted, so that length chain, concentration and threshold are important for sphingolipid function.

### Interplay with ABA signalling pathway

ABA has a key function in cold/drought stress responses. Pioneering work on sphingolipids showed that d18:1‐P and t18:0‐P were rapidly induced by drought and were involved in ABA signalling pathway to control guard‐cell turgor and therefore stomatal aperture (Ng *et al*., 2001; Coursol *et al*., 2003, 2005). This sphingolipid signalling pathway involved Ca^2+^ mobilisation, modification of ion channel activity, and heterotrimeric G‐protein. Consistent with this, AtLCBK1 was reported to be induced by low‐humidity or ABA treatments (Imai & Nishiura, 2005). Moreover, ABA also induces the accumulation of several LCB‐Ps (Guo *et al*., 2012). SPHK1 is an enzyme that phosphorylates d18:1 and t18:0. Stomata of SPHK1‐OE and of *Atspp1* mutant (which accumulates d18:1‐P) displayed a higher sensitivity than WT to ABA (Worrall *et al*., 2008; Nakagawa *et al*., 2012). Therefore, LCB‐P content regulated by LCB kinases and phosphatases plays a key role in the ABA signalling pathway.

### Interplay with phospholipid metabolism

**Figure 3:**
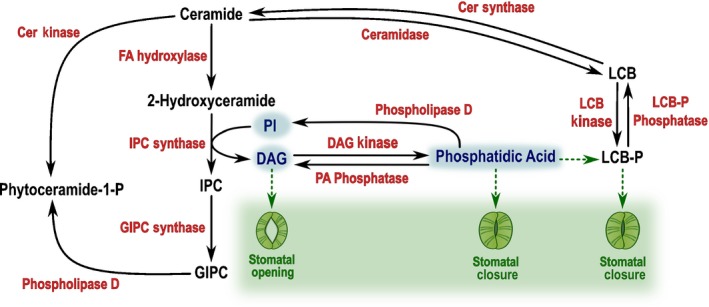
Interplay between sphingolipid and phospholipid metabolisms and their involvement in stomatal aperture. Phospholipid compounds are highlighted in blue. Solid arrows represent enzymatic reactions and dashed arrows indicate a stimulation reaction. Cer, ceramide; DAG, diacylglycerol; FA, fatty acid; GIPC, glycosyl inositol phosphoceramide; IPC, inositol phosphorylceramide; LCB, long‐chain base; LCB‐P, long‐chain base phosphate; PA, phosphatidic acid; PI, phosphoinositol.

Similar to sphingolipids, phosphatidic acid (PA) is considered as a lipid messenger involved in plant response to both biotic and abiotic stress. Like sphingolipids, PA interacts with MPK6 during salt stress response in Arabidopsis (Yu *et al*., 2010) and NADPH oxidase to regulate ROS production during ABA‐regulated stomatal closure (Zhang *et al*., 2009). The PA biosynthetic pathway responds to temperature and salt stress and interacts with sphingosine kinases (Guo *et al*., 2011). Moreover, addition of exogenous PA induced LCB‐P production and LCB‐P levels are diminished in *pld*α*1* in response to ABA (Guo *et al*., 2012). Overexpression of sphingosine kinase increased PA accumulation. Altogether, the crosstalk between PA and sphingolipids should be a critical point to coordinate a stress response that needs to be elucidated (Fig. 3) (Guo & Wang, 2012; Ng & Coursol, 2012). DAG is a by‐product of the IPC synthase and is known to promote stomatal opening (Lee & Assmann, 1991; Peters *et al*., 2010). Although there is no direct evidence for a relationship between sphingolipids and DAG (Fig. 3), lipidome remodelling under stress could yet prove a link.

## Signalling messengers in biotic stress

Biotic stress caused by plant pathogens and insects is a major threat to both plant survival and productivity. Plants have developed a complex set of defences when challenged by pathogens. A successful innate immune response depends on the capability of the plant to recognise its invader and then to translate the different stimuli to an adaptive response. As structural plasma membrane components, sphingolipids are important molecules on the front line of pathogen recognition. Sphingolipid disruption also has an impact on PCD and accumulation of several well known defence molecules (such as ROS, MAPK, and hormones) and sphingolipids therefore act as mediators in the defence signalling cascade.

Very recently, metabolomic profiling identified changes in the sphingolipid pool after exposure to biotic stress. *Xanthomonas campestris* pv*. campestris* infection on *Brassica oleracea* triggered dynamic changes in sphingolipid metabolism including a reduction in the levels of ceramide N‐palmitoylsphinganine (Tortosa *et al*., 2018). Treatment of tomato fruit with the β‐aminobutyric acid elicitor increased the detected levels of ceramide phosphatidylinositol (Wilkinson *et al*., 2017). These metabolomic studies suggested that biotic stresses could impact sphingolipid metabolism.

### Interplay with SA signalling pathway

Genetic and biochemical data suggest that sphingolipids are involved in the regulation of SA levels. Several mutants with altered sphingolipid metabolism displayed higher SA content and activation of SA‐dependent responses. Conversely, both SA and its analogue benzothiadiazole affected sphingolipid metabolism (Shi *et al*., 2015). The Arabidopsis *fah1/2* mutant displayed SA accumulation in addition to an increase in ceramides but moderate changes in LCB accumulation (König *et al*., 2012). This suggests that elevated ceramide levels lead to an increase in salicylate levels. By contrast, the Arabidopsis *loh1* mutant displayed an accumulation of C16‐ceramides but no changes in SA levels (Ternes *et al*., 2011). This discrepancy suggests the sphingolipid trigger for SA accumulation may be more complicated than initially expected. It is noteworthy that these mutants displayed other changes in sphingolipid homeostasis (e.g. *fah1/2* also shows a decrease in glucosylceramides) that maybe have previously been overlooked. The induction of SA could therefore be due to alterations in sphingolipid classes other than LCBs or ceramides. The link between sphingolipid metabolism and SA may rely on MPK6, ROS/NO and/or calcium accumulation but this is still unclear (Sanchez‐Rangel *et al*., 2015). For example, overexpression of LCBK1 in tobacco cell culture triggered the accumulation of ROS in response to cryptogein. Loss of LCBK activity by using inhibitors resulted in a decrease in ROS production in elicited tobacco cells (Coursol *et al*., 2015).

In conjunction with activation of the SA pathway, several studies revealed that plants disrupted in sphingolipid biosynthesis are also affected in their ability to tolerate biotrophic pathogens. Whereas SA is considered essential for resistance to biotrophic and hemibiotrophic pathogens, it has been demonstrated that jasmonic acid (JA) and ethylene (ET) signalling pathways are important for resistance to necrotrophic pathogens in Arabidopsis (Thomma *et al*., 2001; Glazebrook, 2005). In Arabidopsis, it is now acknowledged that SA has a reciprocal antagonistic effect on JA signalling (Glazebrook, 2005). Using *orm1* amiR‐*ORM2* plants, Li *et al*. (2016) demonstrated that the loss of ORM function triggered a constitutive induction of SA‐dependent gene and a tolerance to *Pseudomonas syringae* strain DG3 compared with WT plants. *acd5*,* erh1* (enhancing RPW8‐mediated HR‐like cell death) and *fah1*/*2* mutants also exhibited a constitutive activation of SA pathway and enhanced resistance to powdery mildew. However, they had a similar phenotype to WT after challenge with the hemibiotrophic pathogens *P. syringae* pv. *maculicola* or *Verticillium longisporum* (Wang *et al*., 2008; König *et al*., 2012). Similarly, overexpression of *OsSPL1* in tobacco dramatically reduced SA‐dependent gene expression and increased susceptibility to *P. syringae* pv. *tabaci*. Conversely, *PDF1.2*, a JA‐dependent gene, expression is slightly enhanced (Zhang *et al*., 2014). SA‐dependent pathogenesis‐related (*PR*) gene expressions were constitutively lower in *Atacer‐1* plants compared with WT plants. This profile was similar, but enhanced, when these plants were infected by the *P. syringae* strain DG3. As a consequence, *Atacer‐1* plants were found to be more susceptible to the biotrophic *P. syringae* strain DG3 (Wu *et al*., 2015a). In light of the antagonistic relationship between SA and JA, it would be interesting to analyse SA and JA levels alongside JA‐responsive genes in *Atacer‐1* plants.

Few studies have analysed the role of sphingolipids during plant/necrotrophic pathogen interaction. Tobacco plants in which SPT was silenced accumulated SA, constitutively expressed SA‐induced genes and showed an increased susceptibility to the necrotrophic fungus *Alternaria alternata* f. sp. *lycopersici* (Rivas‐San Vicente *et al*., 2013). Similarly, the SA accumulating *acd5* showed increased susceptibility to *B. cinerea* (Bi *et al*., 2014).

The role of sphingolipid metabolism in response to herbivory has been analysed (Begum *et al*., 2016). Overexpression of OsLCB2a in Arabidopsis led to the accumulation of LCB and ceramides compared with WT. These transgenic plants also displayed increased callose and wax deposition, an induction of SA‐dependent and camalexin‐dependent genes but a reduction of JA‐related genes, and inhibited aphid infestation (Begum *et al*., 2016).

### Interplay with JA signalling pathway

The *Atdpl1* mutant displayed a sensitivity towards the hemibiotrophic bacterium *Pseudomonas syringae* pv. *tomato* but a tolerance when infected by the necrotrophic fungus *Botrytis cinerea* (Magnin‐Robert *et al*., 2015). However, SA levels were similar or even reduced compared with WT, whereas JA levels and JA‐dependent gene expression were higher in the *Atdpl1* infected mutant. This situation suggested a link between the sphingolipid and JA pathway. By using *SPHK1* overexpressing plants, SA production was enhanced in response to FB1 treatment. Conversely *SPHK1*‐KD plants displayed an increase in JA‐related transcripts and metabolites (Qin *et al*., 2017). Therefore, it was suggested that the balance between LCBs and LCB‐Ps modulated by the activity of SPHK1 acted as a signal upstream of the SA/JA signalling pathways during FB1‐induced cell death (Qin *et al*., 2017).

### Interplay with ethylene signalling pathway

It was recently shown that sphingolipid metabolism has connections with not only SA and JA pathways but also with ethylene signalling. Ethylene or its precursor (1‐aminocyclopropane carboxylic acid) inhibits sphingolipid biosynthesis. Mutants disturbed in ethylene biosynthesis or signalling displayed constitutive modifications in sphingolipid content (Wu *et al*., 2015b). For example, *ctr1‐1* mutants, which have enhanced ethylene signalling, contained lower levels of ceramides and hydroxyceramides compared with WT. Some constitutive ethylene response mutants displayed a higher tolerance to FB1, and mutants deficient in ethylene signalling exhibited more sensitivity to FB1, showing that enhanced ethylene signalling rescues FB1‐induced cell death.

## Conclusions and future directions

Over the last few decades we have learned much about the role of sphingolipids during the plant stress response. Functional analyses have demonstrated that sphingolipids are involved in the response to environmental cues. The role of sphingolipids during PCD is well studied. Significant progress has been made but the precise identity of sphingolipids involved in this process is not clearly defined. It is clear that PCD is tightly regulated and further consideration should be given to the different stresses triggering PCD and also the plant species in question. The plasma membrane mediates contact with the environment and is the likely initial source of signal transduction. Recent evidence has shown that GIPC glycosylation involved different regulation processes in the plasma membrane. The composition, distribution and dynamic association of sphingolipids are therefore of high importance for plasma membrane function. It is essential to unravel the dynamic association between sphingolipids, plasma membrane lipids and proteins to better understand the recognition step of the immune response. While a body of evidence has revealed functions for LCBs/LCB‐Ps, ceramides and GIPCs, the roles of GlcCers in plants have yet to be fully investigated, other than the observation that they are essential for normal plant growth and development. The relationship between sphingolipids and SA is long acknowledged and recent studies have shown interconnections with other defence signalling pathways such as JA and ethylene. The regulation of stomatal aperture is of crucial importance during plant defence responses, especially in response to foliar pathogens. ABA‐mediated stomatal closure inhibits pathogen penetration to the apoplast. As the sphingolipid signalling pathway has some interconnections during this process in response to drought stress, the relationship between sphingolipids and ABA in response to foliar pathogens remains to be elucidated.

Despite the range of different structures of sphingolipids and differing physical properties they exhibit, understanding sphingolipid regulation and function is not comprehensive. The interactions with other cellular lipids are also yet to be fully resolved but there are known relationships with several other lipid classes. The wider lipidome is subject to remodelling when the plant is under stress and it is likely that sphingolipids form part of a coordinated response. The mechanisms for action and whether sphingolipids regulate stress responsive gene expression or are themselves regulated by stress responsive transcription factors are not yet fully understood. There is still a gap in understanding the role of sphingolipids in the plant stress response, but the advent of genome editing technology opens the possibility to develop crops with a greater ability to tolerate stress based on the manipulation of their sphingolipid biosynthetic pathway.

## Supporting information

Please note: Wiley Blackwell are not responsible for the content or functionality of any Supporting Information supplied by the authors. Any queries (other than missing material) should be directed to the *New Phytologist* Central Office.

**Table S1** Abbreviations used in this review.
