# Supplementary material for: Sphingolipids: towards an integrated view of metabolism during the plant stress response
Source: New Phytol. 2019 Jul 15;225(2):659–70. doi: 10.1111/nph.15997 (PMC6973233; doi:10.1111/nph.15997)
Supplement: Supplementary file 1 — Table S1 Abbreviations used in this review. [file NPH-225-659-s001.pdf]

New Phytologist **Supporting Information.**

Sphingolipids: towards an integrated view of metabolism during the plant stress response

Eloïse Huby, Johnathan A. Napier, Fabienne Baillieul, Louise V. Michaelson, Sandrine Dhondt-Cordelier

Article acceptance date: 7 June 2019

Table S1 Abbreviations used in this review.

Abbreviation	Full term or name
3-KSR	3-Ketosphinganine reductase
ABA	Abscisic acid
ACBP3	Acyl-coA binding protein 3
ACD5	Accelerated cell death 5
ACER	Alkaline ceramidase
ADS2	Acyl lipid desaturase 2
BI-1	Bax inhibitor 1
Cer	Ceramide
Cer-P	Ceramide phosphate
CerK	Ceramide kinase
coA	Coenzyme A
CPK3	Calcium-dependent protein kinase 3
CTR1	Constitutive triple response 1
DAG	Diacylglycerol
DGDG	Digalactosyldiacylglycerol
DPL1	Dihydrosphingosine phosphate lyase 1
ER	Endoplasmic reticulum
ERH1	Enhancing RPW8-mediated HR-like cell death 1
ET	Ethylene
FAH	Fatty acid hydroxylase
FB1	Fumonisin B1
GA	Gibberellic acid
GlcCer	Glucosylceramide
GCS	Glucosylceramide synthase
GINT1	Glucosamine inositolphosphorylceramide transferase 1
GIPC	Glycosylinositolphosphorylceramide
GMT1	GIPC mannosyl-transferase 1
GONST	Golgi localized nucleotide sugar transporter
HR	Hypersensitive response
IPC	Inositolphosphorylceramide
IPCS	Inositolphosphorylceramide synthase
IPUT1	Inositolphosphorylceramide glucuronosyltransferase 1

iTRAQ	Isobaric tag for relative and absolute quantitation
JA	Jasmonic acid
LCB	Long-chain base
LCB1,2	Subunit of serine palmitoyltransferase 1, 2
LCBK	Long-chain base kinase
LCB-P	Long-chain base phosphate
LOH	Longevity assurance gene one homolog
MAPK	Mitogen-activated protein kinase
MGDG	Monogalactosyldiacylglycerol
NADPH	Nicotinamide adenine dinucleotide phosphate
nCer1	Neutral ceramidase 1
NLP	Necrosis and ethylene-inducing peptide 1-like protein
NO	Nitric oxide
ORM	Orosomucoid like protein
PA	Phosphatidic acid
PCD	Programmed cell death
PI	Phosphoinositol
PLD	Phospholipase D
PR	Pathogenesis-related
RbohB	Respiratory burst oxidase homolog protein B
RNAi	RNA interference
ROS	Reactive oxygen species
RPW8	Resistance to powdery mildew8
S1P	Sphingosine-1-phosphate
SA	Salicylic acid
SBH1,2	Sphingoid base hydroxylase 1,2
SL	Sphingolipid
SLD	Sphingolipid Δ8 long-chain base desaturase
SPHK1	Sphingosine kinase 1
SPP1	Sphingosine-1-phosphate phosphatase 1
SPT	Serine palmitoyl transferase
ssSPT	Small subunit of serine palmitoyltransferase
TAG	Triacylglycerol
VLC	Very long chain
VLCFA	Very long chain fatty acid
WT	Wild type
